# 4D-PET reconstruction using a spline-residue model with spatial and temporal roughness penalties

**DOI:** 10.1088/1361-6560/aabb62

**Published:** 2018-05-04

**Authors:** George P Ralli, Michael A Chappell, Daniel R McGowan, Ricky A Sharma, Geoff S Higgins, John D Fenwick

**Affiliations:** 1 Department of Oncology, University of Oxford, Old Road Campus Research Building, Roosevelt Drive, Oxford OX3 7DQ, United Kingdom; 2 Institute of Biomedical Engineering, Department of Engineering Science, University of Oxford, Old Road Campus Research Building, Roosevelt Drive, Oxford OX3 7DQ, United Kingdom; 3 Radiation Physics and Protection, Oxford University Hospitals NHS Foundation Trust, Churchill Hospital, Oxford OX3 7LE, United Kingdom; 4 NIHR University College London Hospitals Biomedical Research Centre, UCL Cancer Institute, University College London, 72 Huntley Street, London WC1E 6DD, United Kingdom; 5 Institute of Translational Medicine, University of Liverpool, UCD Block, Royal Liverpool University Hospital, Daulby Street, Liverpool L69 3GA, United Kingdom

**Keywords:** dynamic PET, image reconstruction, kinetic modelling, regularization

## Abstract

4D reconstruction of dynamic positron emission tomography (dPET) data can improve the signal-to-noise ratio in reconstructed image sequences by fitting smooth temporal functions to the voxel time-activity-curves (TACs) during the reconstruction, though the optimal choice of function remains an open question. We propose a spline-residue model, which describes TACs as weighted sums of convolutions of the arterial input function with cubic B-spline basis functions. Convolution with the input function constrains the spline-residue model at early time-points, potentially enhancing noise suppression in early time-frames, while still allowing a wide range of TAC descriptions over the entire imaged time-course, thus limiting bias.

Spline-residue based 4D-reconstruction is compared to that of a conventional (non-4D) maximum *a posteriori* (MAP) algorithm, and to 4D-reconstructions based on adaptive-knot cubic B-splines, the spectral model and an irreversible two-tissue compartment (‘2C3K’) model. 4D reconstructions were carried out using a nested-MAP algorithm including spatial and temporal roughness penalties. The algorithms were tested using Monte-Carlo simulated scanner data, generated for a digital thoracic phantom with uptake kinetics based on a dynamic [^18^F]-Fluromisonidazole scan of a non-small cell lung cancer patient. For every algorithm, parametric maps were calculated by fitting each voxel TAC within a sub-region of the reconstructed images with the 2C3K model.

Compared to conventional MAP reconstruction, spline-residue-based 4D reconstruction achieved >50% improvements for five of the eight combinations of the four kinetics parameters for which parametric maps were created with the bias and noise measures used to analyse them, and produced better results for 5/8 combinations than any of the other reconstruction algorithms studied, while spectral model-based 4D reconstruction produced the best results for 2/8. 2C3K model-based 4D reconstruction generated the most biased parametric maps. Inclusion of a temporal roughness penalty function improved the performance of 4D reconstruction based on the cubic B-spline, spectral and spline-residue models.

## 1 Introduction

The uptake of an intravenously injected radiotracer within a patient can be imaged over an extended time-course using dynamic positron emission tomography (dPET). Quantitative data concerning tracer uptake kinetics can be obtained by fitting kinetic models to time-activity curves (TACs) describing the temporal variation of activity within regions of interest (ROIs) drawn on dPET image sequences. These sequences are typically generated by splitting the projection data collected by the scanner into time-frames and reconstructing each frame as an individual image, using either analytical or iterative 2D- or 3D-PET reconstruction algorithms. Image sequences acquired in this way suffer from high levels of noise, due to the limited number of photon counts present in each time-frame and to noise amplification during image reconstruction. This in turn introduces noise and bias into parameter values obtained by fitting kinetic models to the resulting TAC data.

Iterative 4D-PET reconstruction is an alternative methodology, in which images are reconstructed simultaneously for all time-frames, and at each iteration the TAC of every voxel is replaced by the fit to it of a temporally smooth function. While many studies have demonstrated that 4D-PET reconstruction improves the signal-to-noise ratio (SNR) both of reconstructed image sequences and of fitted kinetic parameters, the optimal choice of temporal function remains an open question (Reader and Verhaeghe 2014). A common approach is to use the kinetic model of interest as the temporal function, allowing its kinetic parameter values to be obtained directly from the 4D-PET reconstruction rather than from an additional model fitting step post-reconstruction. This is known as ‘direct’ 4D-PET reconstruction and has been carried out using the spectral model (Matthews *et al* 1997, Meikle *et al* 1998, Reader *et al* 2007) and graphical analysis methods such as the Patlak and Logan plots (Tsoumpas *et al* 2008, Wang *et al* 2008, Cheng *et al* 2014, Karakatsanis *et al* 2016), as well as non-linear compartment models (Kamasak *et al* 2005, Wang and Qi 2012a, Cheng *et al* 2015).

In cancer imaging studies, diverse tissues are often present within the scanner field of view (FOV) and so a wide range of TAC shapes may need to be fitted. Kotasidis *et al* (2014) showed that bias from poorly-fitted regions spatially propagates to well-modelled regions during 4D-PET reconstruction, making it advantageous to use highly flexible functions which can adequately fit TACs in all regions. Non-linear compartment models make specific assumptions about the behaviour of the radiotracer in the tissue being modelled and therefore can only describe a limited range of TAC shapes. Thus a non-linear model designed to accurately describe the uptake kinetics within a tumour may perform poorly in other imaged regions, introducing bias into kinetic parameter estimates even in the well described regions.

Linear kinetic models, which represent each TAC as a weighted sum of pre-defined temporal basis functions, offer considerably more flexibility if the basis functions are well chosen. In situations with diverse kinetics, the spectral model of Cunningham and Jones (1993) and spline functions are often used. Due to their flexibility, spline functions can describe a wide range of TAC shapes well, potentially reducing image noise and bias (Nichols *et al* 2002, Verhaeghe *et al* 2006, Li *et al* 2007, Ralli *et al* 2017), but the fitted parameters of the spline functions themselves do not directly represent physiological information. Furthermore, while fits of the spectral model provide direct estimates of macro-parameters such as the volume of distribution, the micro-parameter values obtained from non-linear compartment model fits are conceptually more directly linked to specific biological processes. Therefore, it may be advantageous to use spline or spectral model-based 4D-PET reconstruction to limit the noise in dPET image sequences, and subsequently analyse the resulting images using the more physiologically-motivated non-linear compartment models.

The temporal basis functions of the spectral model are exponential decays convolved with the arterial input function (AIF), which describes the time-course of radiotracer activity concentration in the arterial blood flowing into a region. TACs vary most rapidly early on during imaging, and so short time-frames are used for the early time-points, making them particularly noisy. Due to their convolution with the AIF, the shapes of spectral basis functions are much more constrained at these early time-points than those of B-splines; but for the same reason they can still describe the early parts of TACs well. Consequently the spectral model may have an advantage at early time-points. On the other hand, B-spline basis functions can describe a wider range of TAC shapes than spectral basis functions, and thus may introduce less bias than the spectral model into the reconstruction process. However, the more flexible splines can also over-fit the data, potentially limiting the degree of noise suppression achievable compared to the spectral model (Ralli *et al* 2017). A temporal model that combines the noise suppressing capabilities of the spectral model, particularly in early time-frames, with the greater flexibility of spline functions might therefore be advantageous.

In this work we propose an alternative linear model, termed the spline-residue model, whose basis functions are B-spline basis functions convolved with the AIF. Like the spectral model, convolution with the input function constrains the spline-residue basis function shapes in early time-frames, potentially enhancing the noise suppression achieved in the early frames by 4D reconstruction. But by using B-splines instead of exponential functions, the spline-residue model can describe a wider range of TAC shapes across the whole imaged time-course than can the spectral model, potentially reducing bias. O’Sullivan *et al* (2009) developed a similar spline-residue model for post-reconstruction non-parametric analysis of radiotracer uptake kinetics in dPET images. To our knowledge, however, use of the model for 4D image reconstruction has not previously been proposed or evaluated.

The spline-residue 4D-PET reconstruction algorithm developed here includes both spatial and temporal roughness penalties. Algorithm performance is evaluated using Monte-Carlo simulated PET detector data collected for a digital phantom, built using TACs obtained from images of a stage IV non-small cell lung cancer (NSCLC) patient injected with the [^18^F]-Fluromisonidazole (FMISO) hypoxia tracer.

Many 4D-PET reconstruction algorithms based on specific linear models have been proposed, but few published studies have inter-compared the performance of different linear models. Furthermore, to our knowledge the performance of direct 4D-PET reconstruction based on a non-linear model has not been compared to that of linear model-based 4D-PET reconstruction followed by kinetics analysis using the same non-linear model. In this study the performance of spline-residue-based 4D-PET reconstruction is compared to that of a conventional (non-4D) MAP reconstruction algorithm, and to 4D-PET reconstruction based on the spectral model, adaptive-knot cubic B-splines and an irreversible two-tissue compartment model commonly used to analyse FMISO dPET data (Wang *et al* 2009, McGowan *et al* 2017). Performance is measured using bias and noise metrics of the reconstructed images, and parametric maps describing voxel-by-voxel compartment model fits to the image data.

## 2 Methods

### 2.1 Nonparametric spline-residue description of dPET TACs

A TAC can quite generally be modelled as the convolution of the AIF with a residue function,

(1) $$f(t)=K\int_{0}^{t}C_{I}(s)R(t-s)ds,$$

where *C_I_* (*t*) is the AIF, *K* is a proportionality constant interpreted as overall flow and *R*(*t*) is the residue function, which describes the fraction of tracer remaining in the region at time *t* after entering it, and so provides information about the kinetics of radiotracer transport and metabolism processes (Gunn *et al* 2001).

The spline-residue model represents the residue function as a weighted sum of *N_S_* cubic B-spline functions, *ζ_k_* (*t*), with a Dirac delta function added to account for the finite blood volume in the region:

(2) $$R(t)=\mu_{0}\delta (t)+\sum_{k=1}^{N_{S}}\mu_{k}\zeta_{k}(t),$$

where *µ_k_* is the coefficient of the *k*th B-spline function *ζ_k_* (*t*), the coefficient *µ*_0_ equals *V_b_/K*, and *V_b_* is the fractional blood volume. Thus from (1) the TAC is given by a weighted sum of spline-residue basis functions *η_l_* (*t*):

(3) $$f(\theta ,t)=\sum_{l=0}^{N_{S}}\theta_{l}\eta_{l}(t),$$

where

(4) $$\eta_{l}(t)=\{\begin{array}{ll} C_{I}(t), & l=0\\ C_{I}(t)⊗\zeta_{l}(t), & l>0 \end{array},$$

⊗ denotes a convolution, and the coefficients *θ_l_* are given by

(5) $$\theta_{l}=\{\begin{array}{ll} V_{b}, & l=0\\ K\mu_{l-1}, & l>1 \end{array}.$$

**Figure 1:**
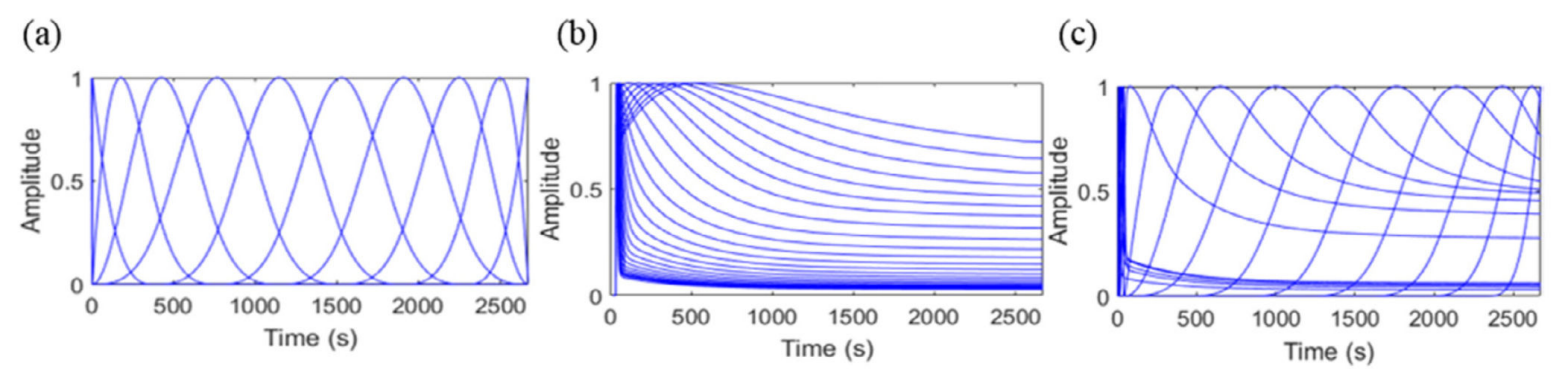
Comparison of typical shapes of normalized (a) cubic B-spline, (b) spectral model and (c) spline-residue model basis functions.

Spline-residue, cubic B-spline and spectral model basis functions are compared in figure 1. The early spectral and spline-residue model basis function are very similar, while the later basis functions are rather different.

### 2.2 Temporally regularized nested-MAP 4D reconstruction algorithm for linear kinetic models

Linear kinetic models represent the number of positron annihilation events in a given voxel *j* at time-frame *m*, *x_jm_*, as a linear combination of pre-defined basis functions:

(6) $$x_{jm}(\theta_{j})=\sum_{k=1}^{N_{B}}B_{km}\theta_{jk},$$

where *N_B_* is the total number of basis functions, *θ_jk_* is the weighting factor of the *k*th basis function *B_k_* (*t*) in voxel *j*, and *B_km_* is given by

(7) $$B_{km}=\int_{t_{ms}}^{t_{mf}}B_{k}(t)\;\text{exp}\;(-\lambda t)\;dt,$$

where *t_ms_* and *t_mf_* are respectively the start and end times of time-frame *m*, and *λ* is the radiotracer decay constant. The basis functions are pre-defined and therefore only the weighting factors ***θ**_j_* need to be calculated when fitting the models.

The expected number of photon counts on detector pair *i* in time-frame *m*, 〈*y_im_*〉, can be estimated as a function of the model parameters using

(8) $$\langle y_{im}(\theta )\rangle =\sum_{j=1}^{N_{V}}P_{ij}\;x_{jm}(\theta_{j})+\varepsilon_{im},$$

where *N_V_* is the total number of voxels, *ε_im_* represents the erroneous counts measured by detector pair *i* in time-frame *m* (random coincidences and scattered photons), *P_ij_* are the elements of the *N_D_ × N_V_* system matrix ***P***, and *N_D_* is the number of detector pairs. The element *P_ij_* represents the probability of a pair of photons originating in voxel *j* being detected by detector pair *i*. The system matrix used here is independent of time, though time-dependent effects such as detector dead-time can be and sometimes are included in the system matrix calculation (Qi *et al* 1998).

Modelling the measured counts *y_im_* as independent Poisson-distributed variables, the log-likelihood function of the measured scanner data *L* (***y***|***θ***) (with a constant term omitted) is

(9) $$L(\text{y}|\theta )=\sum_{m=1}^{N_{T}}\sum_{i=1}^{N_{D}}\left(y_{im}\;\text{ln}\;\left(\langle y_{im}(\theta )\rangle \right)-\langle y_{im}(\theta )\rangle \right),$$

where *N_T_* is the number of time-frames. The most likely parameter values are obtained by iteratively maximizing *L* (***y***|***θ***) with respect to ***θ***. Many basis functions can be included in linear kinetic models, potentially leading to overfitting of the data, and so we modify the objective function to include a temporal regularization term:

(10) Φ(θ)=L(θ|y)−γΓ(θ),

where *γ* is a parameter controlling the trade-off between temporal smoothness and accurate TAC description, and Γ(***θ***) is a temporal roughness penalty defined as

(11) $$\Gamma (\theta )=\sum_{j=1}^{N_{V}}\Lambda (\theta_{j}),$$

with Λ (***θ**_j_*) being the penalty function for voxel *j*.

To efficiently maximize Φ (***θ***) we propose a methodology based on optimization transfer (Lange *et al* 2000), in which determination of ***θ*** at iteration *n* is transferred to a surrogate function *Q* (***θ***|***θ**^n^*) which minorizes the original log-likelihood function:

(12) $$Q(\theta |\theta^{n})⩽\Phi (\theta ),$$

with equality if and only if ***θ*** = ***θ**^n^*. By choosing ***θ***^*n*+1^ as the ***θ*** value maximizing *Q* (***θ***|***θ**^n^*), Lange *et al* (2000) showed that Φ (***y***|***θ***^*n*+1^) ≥ Φ (***y***|***θ**^n^*).

We obtain the surrogate objective function by subtracting *γ*Γ (***θ**_j_*) from the surrogate function proposed by Wang and Qi (2010) for the minorization of *L* (***y***|***θ**^n^*) (with a constant term omitted). Doing so gives:

(13) $$Q\left(\theta |\theta^{n}\right)=\sum_{j}\left(\sum_{i}P_{ij}\right)\left(\left[\sum_{m}\left({\hat{x}}_{jm}^{n+1}\text{ln}\left(x_{jm}\left(\theta_{j}\right)\right)-x_{jm}\left(\theta_{j}\right)\right)\right]-\gamma^{\prime }\text{Λ}\left(\theta_{j}\right)\right),$$

where $\gamma^{\prime }=\frac{\gamma }{\left(\sum_{i}P_{ij}\right)},$ *n* is the current iteration number and ${\hat{x}}_{jm}^{n+1}$ is the image obtained when updating the current image estimates using the MLEM algorithm (Shepp and Vardi 1982):

(14) $${\hat{x}}_{jm}^{n+1}=\frac{x_{jm}\left(\theta_{j}^{n}\right)}{\sum_{i}P_{ij}}\sum_{i}P_{ij}\frac{y_{im}}{\langle y_{im}\left(\theta^{n}\right)\rangle }.$$

Because *Q* (***θ***|***θ**^n^*) is separable in voxels the parameter values $\theta_{j}^{n+1}$ can be obtained using

(15) $$\theta_{j}^{n+1}=\underset{\theta_{j}}{\text{max}}\left[\sum_{m}{\hat{x}}_{jm}^{n+1}\text{ln}\left(x_{jm}\left(\theta_{j}\right)\right)-x_{jm}\left(\theta_{j}\right)-\gamma^{\prime }\text{Λ}\left(\theta_{j}\right)\right].$$

Instead of maximizing (15), we adapt a method proposed by Matthews *et al* (2010) for the un-regularized case, which uses a weighted least squares approach to calculate $\theta_{j}^{n+1}.$ For the temporally regularized case, $\theta_{j}^{n+1}$ is obtained by minimizing the penalized weighted least square error

(16) $$\theta_{j}^{n+1}=\underset{\theta_{j}}{\text{min}}\left[\sum_{m}w_{jm}{\left({\hat{x}}_{jm}^{n+1}-x_{jm}\left(\theta_{j}\right)\right)}^{2}+\gamma^{\prime }\text{Λ}\left(\theta_{j}\right)\right],$$

with weighting factors

(17) $$w_{jm}=\frac{1}{x_{jm}\left(\theta_{j}^{n}\right)}.$$

Wang and Qi (2012a) have noted that monotonic convergence to the maximum-likelihood solution is not guaranteed when the model fitting step is modified to a weighted least squares problem, however convergence has been observed in practice (Matthews *et al* 2010).

Temporal roughness penalties have been used in previous 4D-PET reconstruction studies, but there is no consensus on what form Λ (***θ***_*j*_) should take. Two penalty functions are explored in this work, the first being

(18) $$\text{Λ}\left(\theta_{j}\right)={\left|\theta_{j}\right|}^{2},$$

which corresponds to *L*_2_ regularization, and the second

(19) $$\text{Λ}\left(\theta_{j}\right)=\int_{0}^{T_{scan}}{\left(\frac{\partial^{2}f\left(\theta_{j},t\right)}{\partial t^{2}}\right)}^{2}dt,$$

where *T_scan_* is the dPET scan duration. The second penalty function is often used to fit splines to noisy data and has been applied to spline-based 4D-PET reconstruction (Nichols *et al* 2002, Li *et al* 2007), though not in an optimization transfer framework.

Both penalty functions can be expressed as

(20) $$\text{Λ}\left(\theta_{j}\right)=\theta_{j}^{T}\Omega \theta_{j},$$

where the superscript *T* indicates a matrix transpose. For penalty function (18) **Ω** is the identity matrix ***I***, while for (19) the elements of **Ω**, Ω_*ab*_, are

(21) $$\Omega_{ab}=\int_{0}^{T_{scan}}{\ddot{B}}_{a}\left(t\right){\ddot{B}}_{b}\left(t\right)dt,$$

where *B̈*_*k*_(*t*) is the second-order time-derivative of basis function *k*. Using this notation the cost function in equation (16) can be re-expressed as a Tikhonov regularization problem

(22) $$\theta_{j}^{n+1}=\underset{\theta_{j}}{\text{min}}\left[\sum_{m}w_{jm}{\left({\hat{x}}_{jm}^{n+1}-x_{jm}\left(\theta_{j}\right)\right)}^{2}+\gamma^{\prime }\theta_{j}^{T}\Omega \theta_{j}\right],$$

and solved using the equation (Tikhonov *et al* 1995)

(23) $$\theta_{j}^{n+1}={\left({\text{B}}^{T}\text{WB}+\gamma^{\prime }\Omega \right)}^{-1}{\text{B}}^{T}\text{W}{\hat{\text{x}}}_{j}^{n+1},$$

where ***B*** has the *B_km_* elements defined in equation (7), ***W*** is the diagonal matrix *diag* (*w*_*j*1_, *w*_*j*2_, … , *w*_*jN_T_*_) and ${\hat{\text{x}}}_{j}^{n+1}$ is an *N_T_ ×* 1 vector with *m*th element ${\hat{x}}_{jm}^{n+1}.$

To determine the best *γ′* to use in (22) for voxel *j*, $\gamma_{j}^{\prime },$ a range of *γ′* values can be defined in advance, and the one producing the fit with the lowest generalized cross validation (GCV) score (Wahba 1990) taken as optimal for this voxel:

(24) $$\gamma_{j}^{\prime }=\underset{\gamma \prime }{\text{min}}\;\left[GCV\left(\gamma^{\prime }\right)\right]=\underset{\gamma \prime }{\text{min}}\;\left[\frac{\sum_{m}w_{mj}{\left({\hat{x}}_{jm}^{n+1}-x_{jm}\left(\theta_{j}^{n+1}\right)\right)}^{2}}{{\left(\text{Tr}\left(I-B{\left(B^{T}WB+\gamma^{\prime }\Omega \right)}^{-1}B^{T}W\right)\right)}^{2}}\right],$$

where Tr(…) denotes the matrix trace, and the denominator of (24) corresponds to the effective degrees of freedom. The model fitting step is much faster than the image update step, and so selection of the *γ′* value for each voxel TAC in this semi-automatic manner does not greatly slow down the reconstruction.

Spatial regularization can be built into the reconstruction by replacing the image update step in equation (14) with a corresponding step from an iterative 2D- or 3D-maximum *a posteriori* (MAP) algorithm. These image updates are designed to maximize objective functions of the form *L* (***x***|***y***) – *βU*(***x***) with respect to the image ***x***, where *β* is a tunable parameter controlling the trade-off between resolution and noise, and *U* (***x***) is a concave function designed to penalize rough images,

(25) $$U\left(\text{x}\right)=\frac{1}{4}\sum_{j}\sum_{k\in {𝒩}_{j}}z_{jk}\psi \;\left(x_{j}-x_{k}\right),$$

where ${𝒩}_{j}$ is the set of nearest neighbours of voxel *j* and *z_jk_* is a weighting factor equal to the normalized inverse distance between voxels *j* and *k* (Wang and Qi 2012b). Here we use the Lange function (Lange 1990)

(26) $$\psi \left(\xi \right)=\left(\frac{\left|\xi \right|}{\delta }-\text{ln}\left(1+\frac{\left|\xi \right|}{\delta }\right)\right)\delta ,$$

which contains a further smoothing parameter *δ*, and achieves good noise suppression in fairly uniform regions, while preserving edges better than the more widely used quadratic function, *ψ* (*ξ*) = *ξ*^2^ (Lange 1990).

The proposed temporally regularized 4D-PET reconstruction algorithm for linear kinetic models, subsequently referred to as nested-MAP reconstruction, can be summarized as follows.

1. Start with an initial dPET image sequence estimate, in this work a sequence of uniform images with the radioactivity concentration in each voxel set to 100 Bq/cc.
2. Update each image with one iteration of the MAP algorithm. Here MAP updates were performed via algorithm 1 of Wang and Qi (2012b) using their pixel-based rather than patch-based approach. Values of *β* and *δ* are pre-selected.
3. Fit a temporal model to each voxel TAC via (23), using either a fixed value of *γ* or a range of *γ* values and subsequently selecting the best value via GCV.
4. Return to step 2 using the image voxel values predicted by the fitted model, $x_{jm}\left(\theta_{j}^{n+1}\right),$ as the seed for next MAP update, and continue for either a fixed number of iterations or until the images have converged.

### 2.3 Digital phantom simulations

The 4D-XCAT2 digital phantom package (Segars *et al* 2010) was used to simulate a single slice of an NSCLC patient injected with the FMISO hypoxia tracer. Tracer activity concentrations in different regions and time-frames were chosen to match smoothed TACs taken from a clinical FMISO-dPET image of a patient with stage IV NSCLC. Specifically, lung and bone TACs were obtained from spherical ROIs of 3 cm diameter placed in healthy lung and spine regions in the real patient image, and an AIF TAC was taken from a cylindrical ROI of diameter 10 mm located in the centre of the descending aorta on five consecutive PET axial slices. Tumour TACs were obtained from irregularly-shaped ROIs considered to contain hypoxic and normoxic tumour tissue. The ROIs were drawn by an experienced radiologist and checked by a second radiologist.

**Figure 2:**
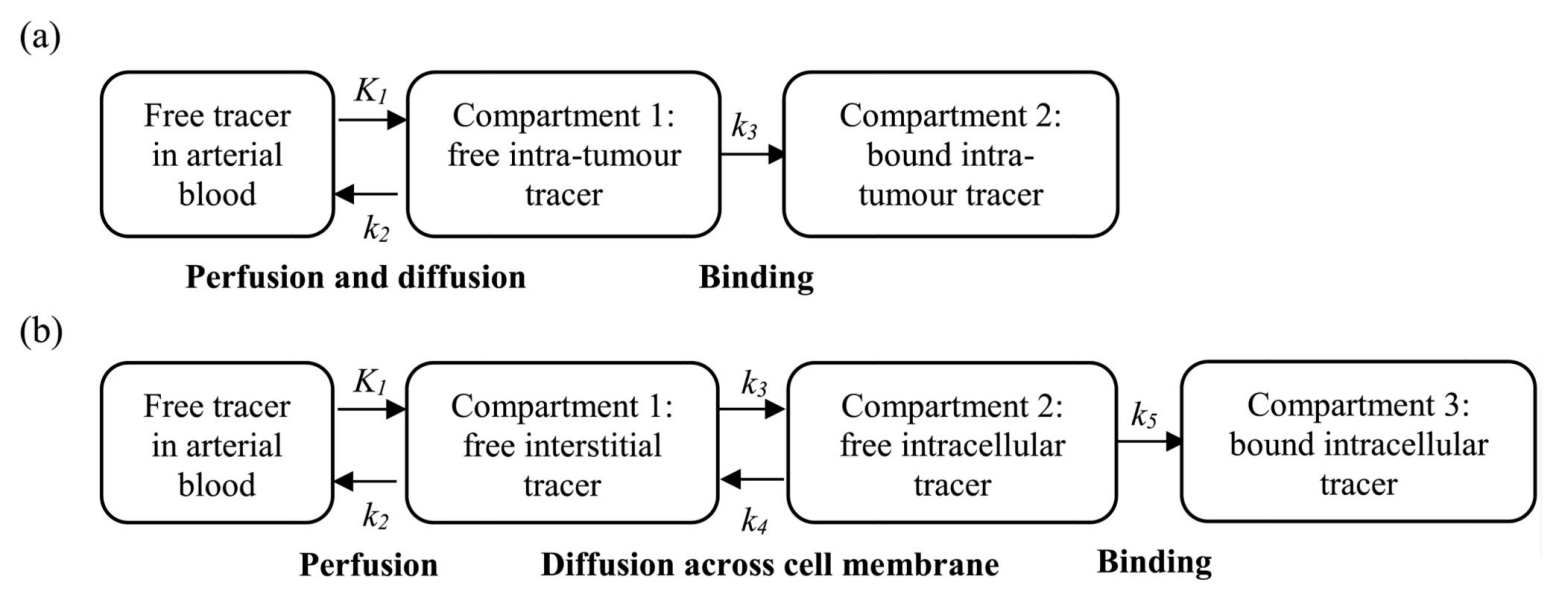
Schematic diagrams showing irreversible (a) two and (b) three tissue compartment models of FMISO uptake. Flows between compartments are defined by rate-constants (*k* values) and compartment tracer concentrations.

For smoothing, each TAC except the AIF was fitted with cubic splines, adaptively placing the knots according to the algorithm proposed by Ralli *et al* (2017), and with irreversible two- and three-tissue compartment models having 3 and 5 rate-constants respectively. These compartment models are schematically drawn in figure 2 and subsequently referred to as 2C3K and 3C5K. The AIF TAC was fitted with the phenomenological three-exponential model of Feng *et al* (1993) alone. Weighted least squares was used for all fitting, with the weighting factors

(27) $$w_{m}=\frac{\Delta T_{m}e^{-\lambda T_{m}}}{a_{m}},$$

where *a_m_* is the decay-corrected average activity concentration within the region during frame *m*, Δ*T_m_* is the frame duration, and *T_m_* is the mid-point of the *m*th frame (Chen *et al* 1991). Compartment model fitting was carried out using the Levenberg–Marquart algorithm, available in the MATLAB optimization toolbox (Mathworks).

Model fit quality was assessed for each TAC using leave-one-out cross-validation, calculating the weighted residual sum of squares (RSS) error of the fit ith the weighting factors defined in (27). The Wald-Wolfowitz runs test was used to check whether any significant structure remained in the residuals of each model fit at the 5% significance level. Of the models that passed the runs test for a given patient TAC, the best model fit was taken to be the one with the lowest weighted RSS value.

Two patient phantoms were created with identical spatial geometries and voxel dimensions of 3.1 × 3.1 × 2.0 mm^3^. Lung, bone, blood, normoxic and hypoxic tumour regions were filled with noise-free ground-truth activity concentrations that varied with time according to model fits to the TACs obtained from the corresponding regions in the patient image. For the first ‘realistic’ phantom the best model fits to the different TACs were used, while for the second ‘simplified’ phantom, 2C3K model fits were used instead. The fitted curves were binned into a (1 × 30 s, 6 × 5 s, 6 × 20 s, 7 × 60 s, 10 × 120 s, 3 × 300 s) time-frame sequence followed by two additional 600 s frames at 2 and 4 h post-injection. This frame sequence matches the dynamic imaging protocol of the clinical dPET scan from which the phantom TACs were derived: following this protocol, the patients were injected with the FMISO tracer 30 s into scanning.

**Figure 3:**
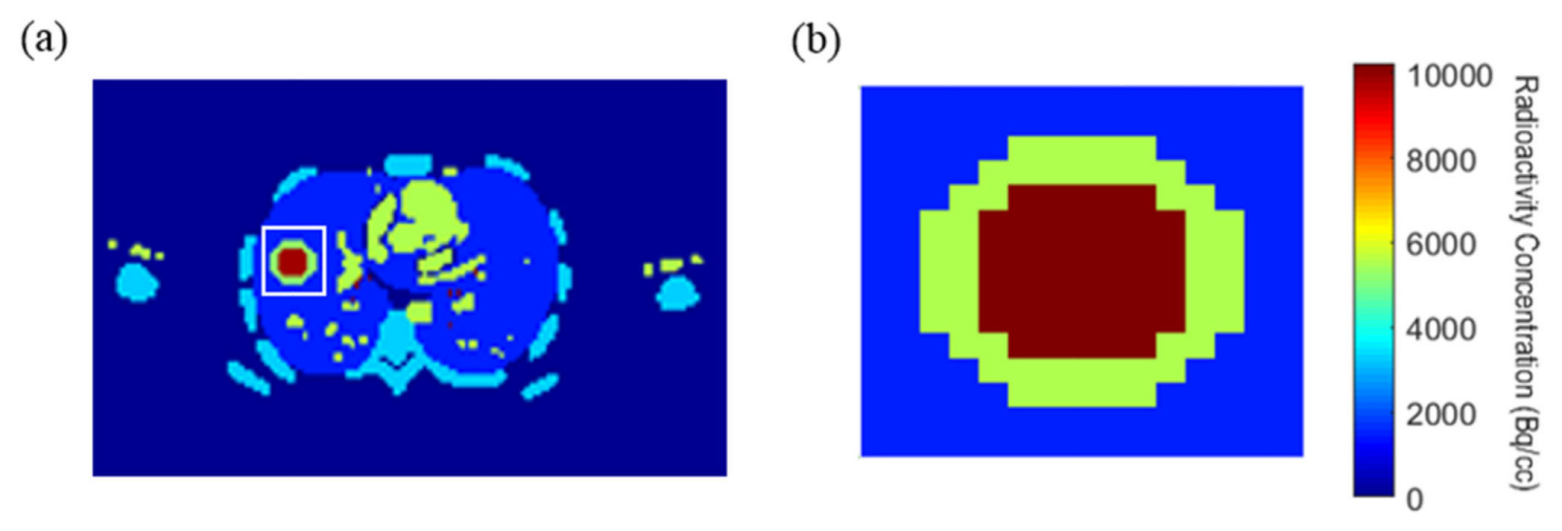
(a) Image of the final time-frame of the realistic phantom, the white box marking the sub-region (b) of the phantom for which parametric maps were calculated.

To illustrate the phantom geometry, an image of the final time-frame of the realistic phantom is shown in figure 3(a). We have used the phantoms to study the performance of linear model-based versus 2C3K-based 4D-PET reconstruction when all the underlying TACs take realistic shapes (realistic phantom), and when they are all described by fits of the 2C3K model (simplified phantom).

dPET sinograms representative of those produced by an mMR PET-MR scanner (Siemens Healthcare, Erlangen, Germany) were generated for both phantoms using the PET-SORTEO Monte-Carlo simulation package (Reilhac *et al* 2004), which has been validated for the Siemens mMR scanner (Reilhac *et al* 2016). Fifty noise realizations of dynamic-PET sinogram data were generated for each phantom, including effects of scattered photons, random co-incidences and attenuation. No patient motion was simulated, the focus of the current study being to evaluate the effectiveness of noise suppression using 4D-PET reconstruction. On average, the total number of counts in each noise realization was approximately 3.5 million for both single-slice phantoms studied.

### 2.4 Image reconstruction

Attenuation and normalization correction sinograms were obtained respectively from an attenuation map of the patient phantom, and from simulated detector counts generated using PET-SORTEO for a 20 min scan of a cylindrical phantom containing a uniform activity concentration. The attenuation and normalization corrections were then modelled as part of the system matrix. Numbers of scattered photons and random coincidences were estimated using the single-scatter simulation algorithm (Watson 2000) and a delayed co-incidence window respectively.

From each simulated realization of FMISO patient phantom PET scanner data, dPET image sequences were reconstructed in 3.1 × 3.1 × 2.0 mm^3^ voxels using both conventional MAP and 4D nested-MAP algorithms, running each algorithm for 30 iterations. Nested-MAP 4D reconstructions were performed using the non-linear 2C3K model, and the linear adaptive-knot cubic B-spline, spectral and spline-residue models. For each linear model, reconstructions were carried out using the temporal regularisation penalties of equations (18) and (19), and with no temporal regularisation. For 2C3K model-based reconstructions no temporal regularization was used because this model is much more constrained than the linear ones. For nested-MAP reconstruction based on the 2C3K model, the non-linear model fitting step was performed using the Levenberg-Marquardt algorithm instead of (23). Spectral, spline-residue and 2C3K model-based 4D reconstructions require image-derived or blood-sampled AIFs, which were obtained here by fitting the three-exponential model of Feng *et al* (1993) to TACs obtained from conventionally (not 4D) reconstructed MAP images, for ROIs placed in the left ventricle.

Spectral model-based reconstructions were carried out using 100 basis functions with exponential decay constants spaced logarithmically between 1.1 × 10^−4^ s^−1^ (the decay constant of ^18^F) and 0.01 s^−1^. For spline-based reconstructions, voxel-specific knot locations were selected using the adaptive-knot placement algorithm proposed by Ralli *et al* (2017), which for cubic splines places knots along equal segments of the integral of the 4th root of the 4th derivative of a TAC according to theorem XII.5 of De Boor (1978). For each voxel 11 free knots were positioned by applying the algorithm to the TAC obtained for that voxel from conventional MAP-reconstructed images.

Basis functions for the spline-residue model were obtained by placing 4 knots at the beginning and end of each TAC, as well as the point where the TAC starts to rise, to handle discontinuities, and positioning an additional 6 uniformly spaced knots between the initial rise and end points of the TAC. The B-splines associated with these knots were convolved with the AIF to calculate the spline-residue basis functions. Preliminary work fitting the spline-residue model to synthetic noisy FMISO TACs led to the choice of 6 additional knots, this number generating fits that best matched the ground-truth. Uniformly spaced knots performed well, perhaps because the residue function for a given TAC varies considerably less than the TAC itself.

The spatial regularization parameters *β* and *δ*, defined in (26), were both set to 0.1, a choice that produced the best contrast-to-noise ratio in images of digital phantom similar to the NEMA image quality phantom (National Electrical Manufacturers Association 2013) reconstructed using the MAP algorithm from simulated PET detector data generated with PET-SORTEO.

For each linear model and temporal roughness penalty used in the 4D reconstruction algorithms, the range of *γ′* values {*γ′* = 0.001, 0.002, …, 0.01} all produced good fits to TACs from conventional MAP-reconstructed patient phantom images. At each iteration of the temporally regularized nested-MAP reconstructions, therefore, the *γ′* value used for each voxel was individually selected from those ten as the one that minimized the GCV score of the model fit to that voxel’s TAC, as described in section 2.2.

### 2.5 Image analysis

#### 2.5.1 Image quality metrics

To characterize the accuracy of the reconstructed images, the average absolute bias of imaged activity concentrations over the scan time-course was calculated for every voxel *j*:

(28) $${\left[\text{Image}\;\text{Bias}\right]}_{j}=\frac{1}{T_{scan}}\sum_{m}\Delta T_{m}\left|{\bar{a}}_{jm}-a_{jm}^{true}\right|,$$

where *ā_jm_* is the mean activity concentration in voxel *j* at time-frame *m* in all 50 repeat image sequences, and $a_{jm}^{true}$ is the true activity concentration.

The noise in each voxel *j* at every time-frame *m* was calculated using the weighted standard deviation *σ*_*w*,*jm*_

(29) $$\sigma_{w,jm}={\left(\frac{\sigma_{jm,\;measured}^{2}\Delta T_{m}}{a_{jm}^{true}}\right)}^{\frac{1}{2}},$$

where $\sigma_{jm,measured}^{2}$ is the variance amongst the 50 repeat *a_jm_* values, and the weighting factors $a_{jm}^{true}/\Delta T_{m}$ are based on the dynamic-PET noise model of Chen *et al* (1991) and nominally account for intrinsic variations in noise between frames. Then the average noise for a given voxel *j* was calculated across all *N_T_* time-frames

(30) $${\left[\text{Image}\;\text{Noise}\right]}_{j}=\sigma_{wj}=\frac{1}{N_{T}}\sum_{m=1}^{N_{T}}\sigma_{w,jm}.$$

Overall bias and noise were characterised as mean absolute bias and mean image noise (〈*σ_w_*〉) averaged over all voxels within the patient (the whole patient region). The same measures were also averaged over the tumour region alone, usually the primary focus of oncological dPET studies. Normalised mean absolute bias and 〈*σ_w_*〉 values were expressed as percentages of the mean ground-truth activity averaged over all time-frames and all voxels in the whole patient or tumour regions. To facilitate algorithm inter-comparison, normalised mean image noise and absolute bias values were computed at every iteration of all 4D reconstructions. Corresponding values were also calculated for just the first 120 s of the scans, to assess algorithm performance at early time-points.

To assess the convergence of the reconstruction process, the mean square error (MSE) was calculated for each image voxel *j* at time-frame *m* and iteration *n* of the nested-MAP reconstructions

(31) $${\text{MSE}}_{jm}^{n}=\frac{1}{N_{r}}\sum_{k=1}^{N_{r}}{\left(a_{jm}^{true}-a_{jm,k}^{n}\right)}^{2},$$

where *N_r_* is the number of noise realizations. At each iteration, a weighted sum of ${\text{MSE}}_{jm}^{n}$ over all time-frames was calculated for every individual voxel, and these values were summed over all image voxels to create a single total MSE measure, TMSE:

(32) $${\text{TMSE}}^{n}=\sum_{j=1}^{N_{V}}\sum_{m=1}^{N_{T}}\frac{\Delta T_{m}{\text{MSE}}_{jm}^{n}}{a_{jm}^{true}},$$

where *N_V_* is the number of image voxels. To check that the images did not change substantially after the first 30 reconstruction iterations explored throughout most of this study, each nested-MAP reconstruction was run for a further 10 iterations. Fractional changes in TMSE from one iteration *n* to the next *n* + 1 were calculated as

(33) $$\left[\text{fractional}\;\text{TMSE}\;\text{change}\;\text{at}\;\text{iteration}\;n\right]=\frac{{\text{TMSE}}^{n}-{\text{TMSE}}^{n+1}}{{\text{TMSE}}^{n}},$$

and plotted as a function of iteration number for *n* = 1 to *n* = 39, positive TMSE changes corresponding to reductions in the total error, and negative changes to increases. Additionally, the fractional change between iterations 30 and 40 was calculated as

(34) $$\left[\text{fractional}\;\text{TMSE}\;\text{change}\;\text{at}\;\text{iteration}\;30/40\right]=\frac{{\text{TMSE}}^{30}-{\text{TMSE}}^{40}}{{\text{TMSE}}^{30}}.$$

#### 2.5.2 Parametric map quality metrics

FMISO uptake kinetics are often determined using the 2C3K model (Wang *et al* 2009, McGowan *et al* 2017). Following this approach, we have fitted the 2C3K model to voxel TACs obtained from all the image sequences reconstructed using the different algorithms. The voxels studied were those lying within the phantom sub-volume shown in figure 3(b), which contains the tumour-like region and surrounding lung and is therefore of the greatest interest. Then we determined the accuracy and precision of uptake kinetics as characterised by the 2C3K model fits to the reconstructed images versus fits of the same model to the ground-truth phantom TACs, studying the 2C3K model rate-constants shown in figure 2(a) together with the flux constant

(35) $$k_{flux}=\frac{K_{1}k_{3}}{k_{2}+k_{3}}.$$

The bias and noise in fitted values of each parameter *q* = *K*_1_, *k*_2_, *k*_3_, *k_flux_* were calculated for each voxel *j* lying within the image sub-volume:

(36) $${\left[\text{Parameter}\;\text{Bias}\right]}_{j}={\bar{q}}_{j}-q_{j}^{true},$$

(37) $${\left[\text{Parameter}\;\text{Noise}\right]}_{j}=\sigma_{qj},$$

where $q_{j}^{true}$ is the ground-truth value of kinetic parameter *q* in voxel *j*, *q̄_j_* is the mean of the *q_j_* values obtained for voxel *j* from each of the 50 reconstructed image sequences, and *σ_qj_* is the standard deviation of these 50 *q_j_* values. Then the [Parameter Bias]_*j*_ and [Parameter noise]_*j*_ measures were averaged over all the voxels within the analysed phantom sub-volume to characterize the overall performance of each reconstruction algorithm. These metrics were also averaged over a region containing hypoxic tumour alone, to assess the performance of the algorithms specifically within the tumour region.

## 3 Results

### 3.1 Selection of fitted TACs for the realistic digital phantom

**Figure 4:**
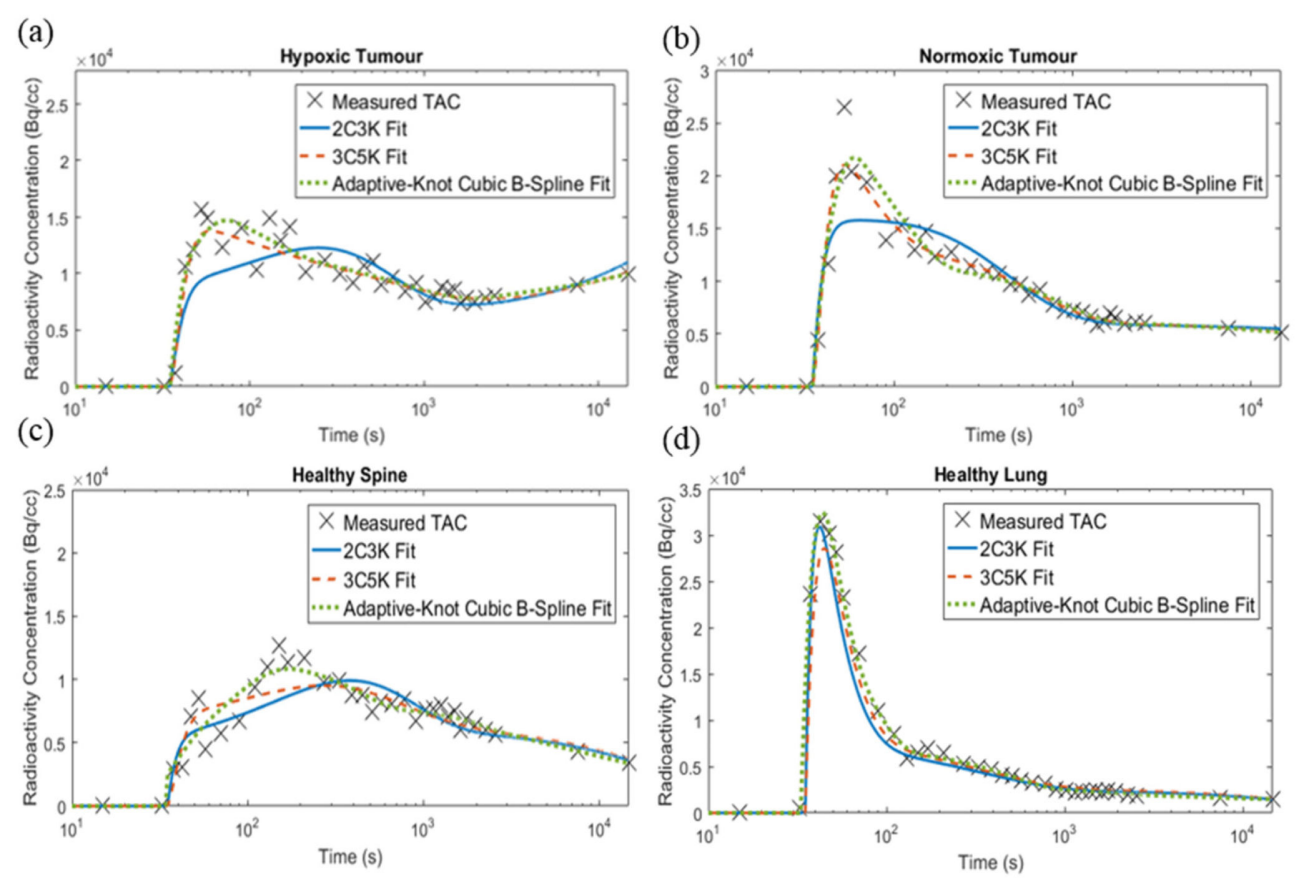
2C3K, 3C5K and adaptive-knot cubic B-spline fits to the TACs obtained from (a) hypoxic tumour, (b) normoxic tumour, (c) healthy lung and (d) healthy spine ROIs in an FMISO dPET image sequence of a stage IV NSCLC patient.

Figure 4 shows fits of the 2C3K, 3C5K and cubic spline models to the real TAC data obtained from normoxic and hypoxic tumour and healthy lung and spine regions of the imaged NSCLC patient. Runs test results and leave-one-out cross-validation weighted RSS scores are listed in table 1.

Fits of the adaptive-knot spline and 3C5K models had the lowest cross-validation scores for the healthy tissue and tumour regions respectively, and were therefore used to represent the ground-truth TACs for these regions in the realistic phantom. All the model fits used in the realistic phantom passed the runs test. The worst leave-one-out cross-validation scores were obtained for the 2C3K model fits, which only passed the runs test for the hypoxic tumour region.

### 3.2 Image quality metrics

**Figure 5:**
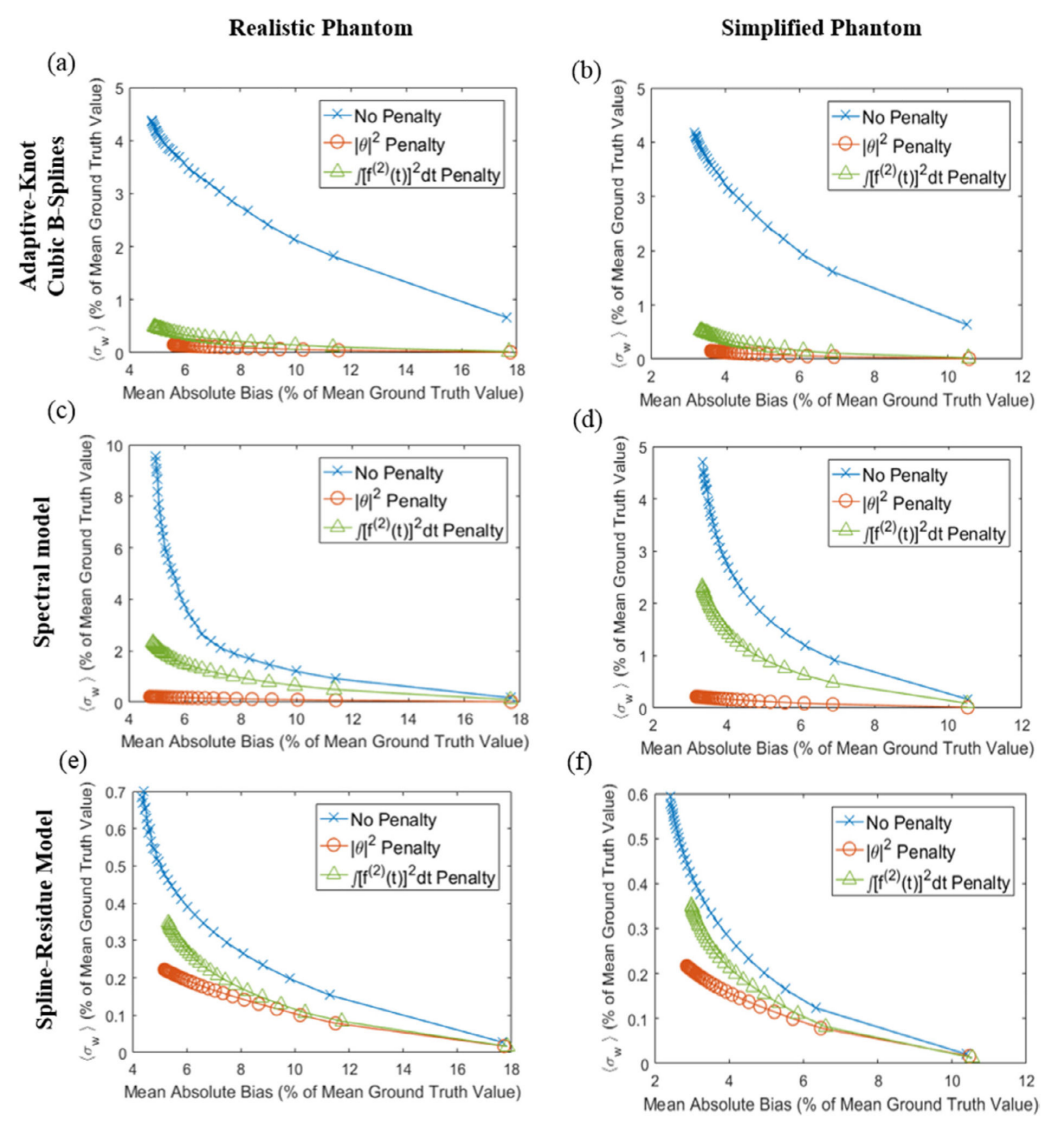
Comparison of image reconstruction results obtained for the realistic (first column) and simplified (second column) phantoms using 4D algorithms based on the (a) and (b) cubic splines, (c) and (d) spectral and (e) and (f) spline-residue models with different temporal roughness penalties. The plots show image noise (weighted standard deviation, *σ_w_*) versus bias at each reconstruction iteration, and have different scales to allow clear visualisation of performance differences between the different penalty functions.

Iteration-by-iteration plots of image bias versus noise, averaged over all time-frames and patient voxels, are shown in figure 5 for realistic and simplified phantom image sequences reconstructed using temporally regularized nested-MAP 4D algorithms based on the cubic spline, spectral and spline-residue linear models. Each plot compares results obtained for one phantom and one reconstruction algorithm using either no temporal roughness penalty or the penalty functions of equations (20) or (21).

For spectral and spline-residue-based 4D reconstructions, the |***θ***|^2^ temporal roughness penalty of equation (18) produced substantially less noisy images than the other penalty options, at similar levels of bias, and was therefore considered the best penalty function for these algorithms. For cubic spline-based 4D reconstructions, however, the integrated square derivative penalty function of equation (19) was viewed as the best penalty function, since it produced the least biased images at noise-levels only slightly higher than obtained using the |***θ***|^2^ penalty.

**Figure 6:**
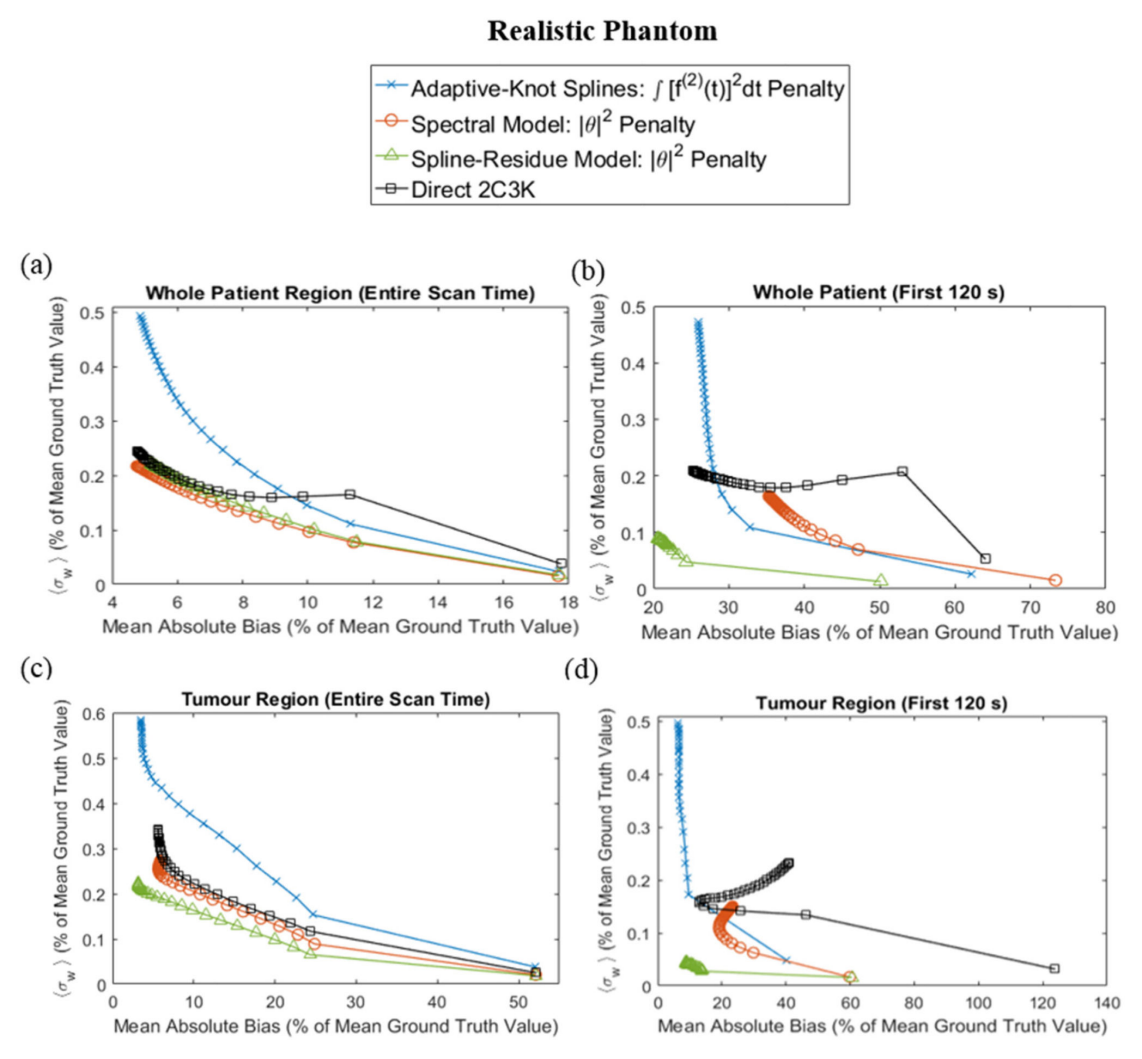
Comparison of image quality metrics for 4D reconstructions of the realistic phantom based on linear models (with their optimal temporal roughness penalty) and on the 2C3K model. The noise and bias metrics are averaged over the whole patient (a) and (b) and tumour (c) and (d) phantom regions, and over the entire scan time (a) and (c) and first 120 s (b) and (d).

In figure 6, noise is plotted against bias for images of the realistic phantom reconstructed using each linear model-based 4D algorithm and its associated optimal temporal roughness penalty, and using with the 2C3K-based 4D algorithm. Separate plots are shown for noise and bias measures averaged over the whole patient or the tumour regions, and averaged over the whole scan time or just the first 120 s. Image quality curves for reconstructions based on the spline-residue model were substantially better than those obtained for reconstructions based on cubic splines or the 2C3K model. And at early time-points and within the tumour region, the image quality curves for the spline-residue-based algorithm were also better than those for reconstructions based on the spectral model. However, the spectral model had a slight edge when the image quality metrics were averaged across the whole phantom and scan duration.

**Figure 7:**
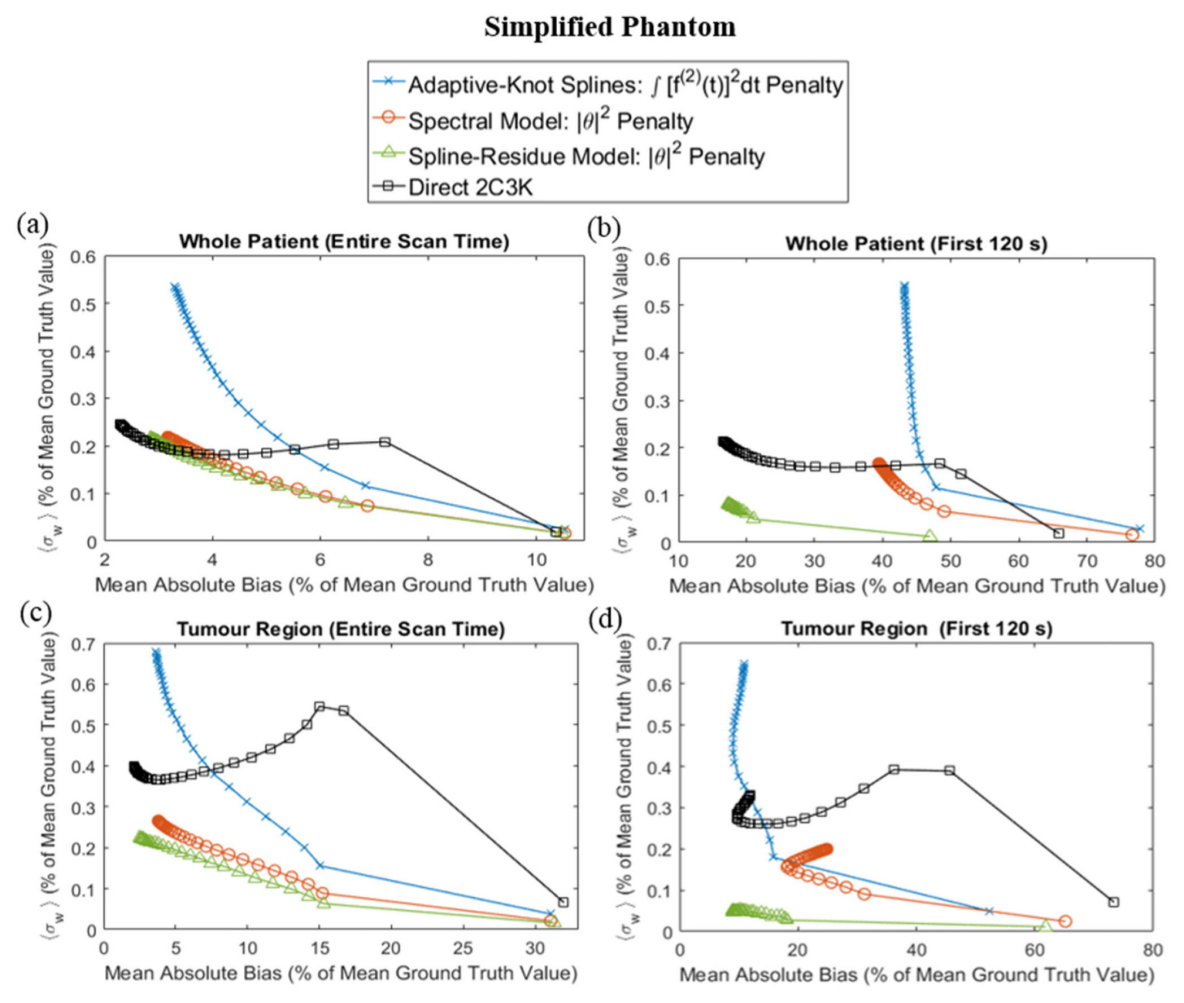
Comparison of image quality metrics for 4D reconstructions of the simplified phantom based on linear models (with their optimal temporal roughness penalty) and on the 2C3K model. The noise and bias metrics are averaged over the whole patient (a) and (b) and tumour (c) and (d) phantom regions, and over the entire scan time (a) and (c) and first 120 s (b) and (d).

Corresponding data are shown in figure 7 for reconstructions of the simplified phantom. For this phantom the spline-residue model produced slightly better results than the spectral model when considered across the whole phantom and scan duration; but 4D reconstructions based on the 2C3K model achieved the lowest bias values, unsurprisingly since the phantom kinetics are 2C3K-based.

**Figure 8:**
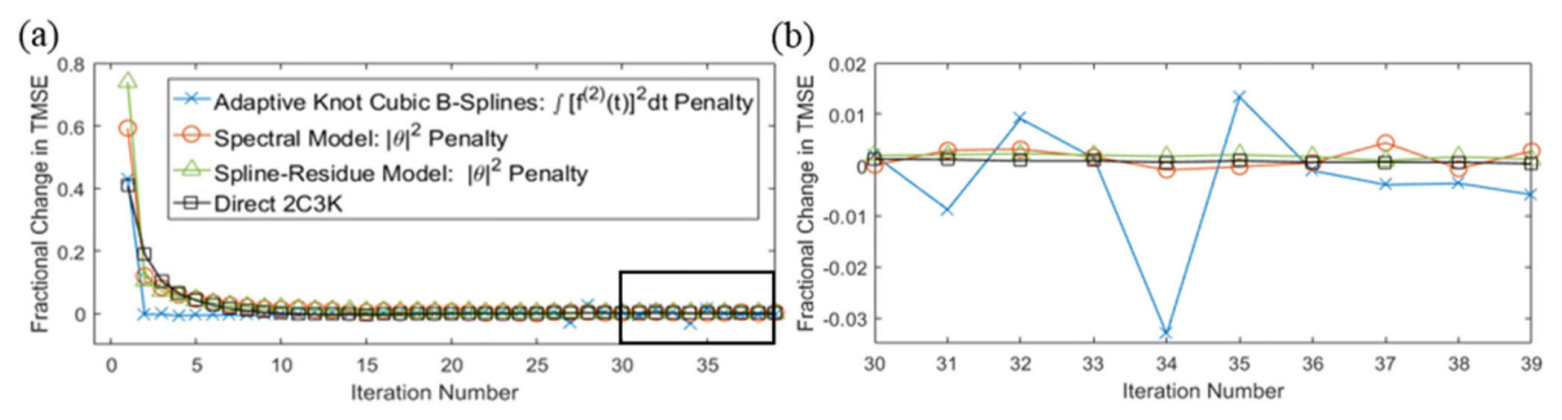
(a) Fractional iteration-to-iteration change in total mean square error (TMSE) versus iteration number, plotted for 2C3K and linear model-based nested-MAP reconstructions of the realistic phantom, the linear model-based reconstructions using the optimal temporal roughness. (b) A magnification of the rectangular region outlined in (a).

Figure 8 shows fractional changes in TMSE as a function of iteration number for the 2C3K- and linear model-based reconstructions of the realistic phantom, using the optimal temporal roughness penalties for the linear models. After 30 iterations the fractional change in TMSE per iteration was very small for reconstructions based on the spectral, spline-residue and 2C3K models. Total fractional changes in TMSE between iterations 30 and 40, calculated with (34), were 0.013 for the spectral model, 0.017 for the spline-residue model, 0.007 for the 2C3K model and −0.030 for cubic splines. Thus continuing reconstruction beyond 30 iterations led to small improvements at best, and in the case of the spline-based reconstruction a 3% worsening in TMSE, most likely due to noise amplification at the later iterations.

### 3.3 Parametric maps

**Figure 9:**
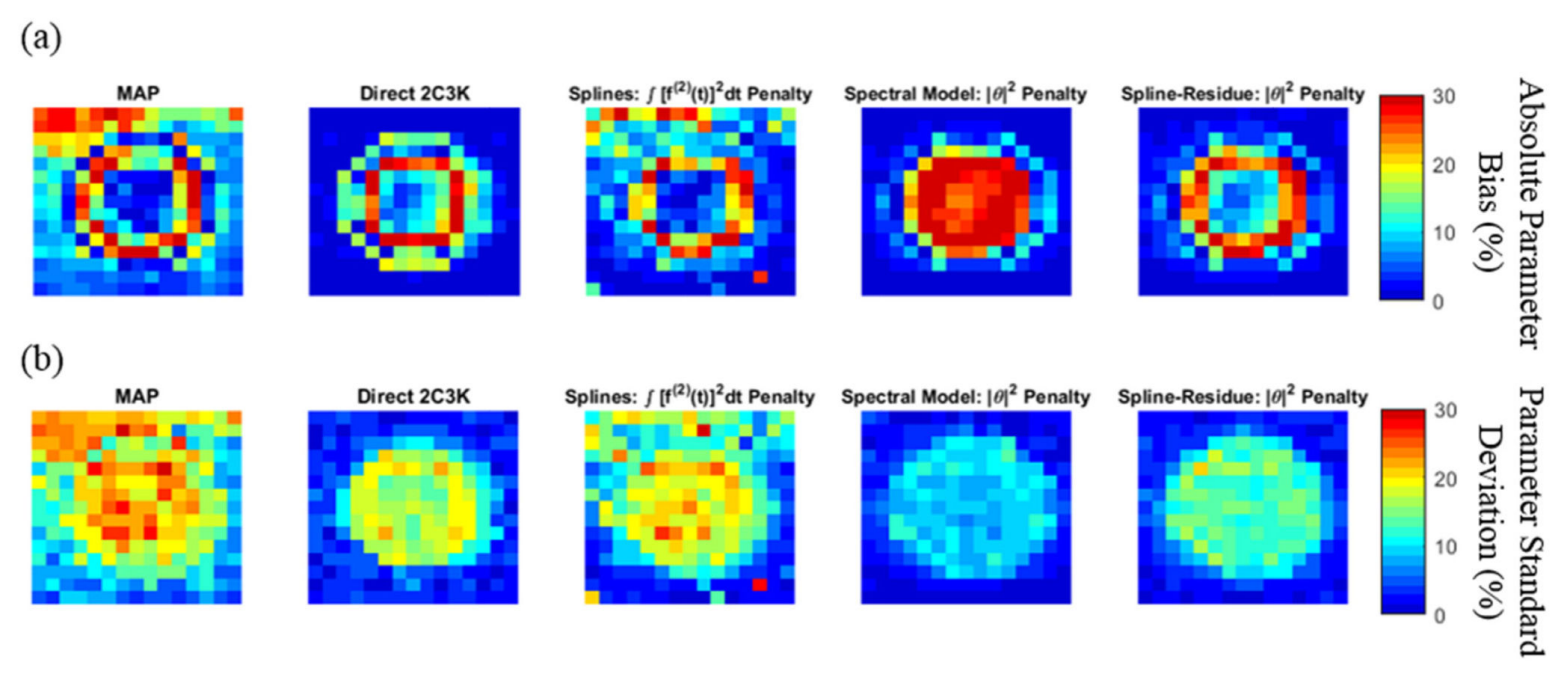
Voxel-by-voxel plots of (a) the absolute bias and (b) the noise (standard deviation) in the *k_flux_* parametric maps of the sub-region of the realistic phantom obtained from image sequences reconstructed using the conventional (non-4D) MAP algorithm, as well as using the 2C3K and linear model-based nested-MAP reconstructions (with their optimal temporal roughness penalties). Bias and standard deviation values are expressed as percentages of the average ground-truth *k_flux_* value across the phantom sub-region.

Figure 9 shows voxel-by-voxel spatial plots of absolute bias and noise in *k_flux_* parametric maps of the realistic phantom sub-region shown in figure 3(b), obtained from image sequences reconstructed using the conventional (non-4D) MAP algorithm and nested-MAP 4D algorithms based on the 2C3K model and linear models used with their optimal temporal roughness penalties. The voxel-by-voxel noise and bias plots calculated for the simplified phantom were similar. The results in figure 9 are summarized in table 2, which lists the signed bias and noise in the *k_flux_* parametric maps averaged over the entire phantom sub-region. In both figure 9 and table 2 the bias and noise are expressed as percentages of the ground-truth *k_flux_* value, averaged over the entire sub-region. Of all the algorithms compared, spline-residue model-based reconstruction achieved the lowest average bias and second lowest noise levels in the *k_flux_* parametric maps, the spectral model-based algorithm achieving lower noise but greater bias.

**Figure 10:**
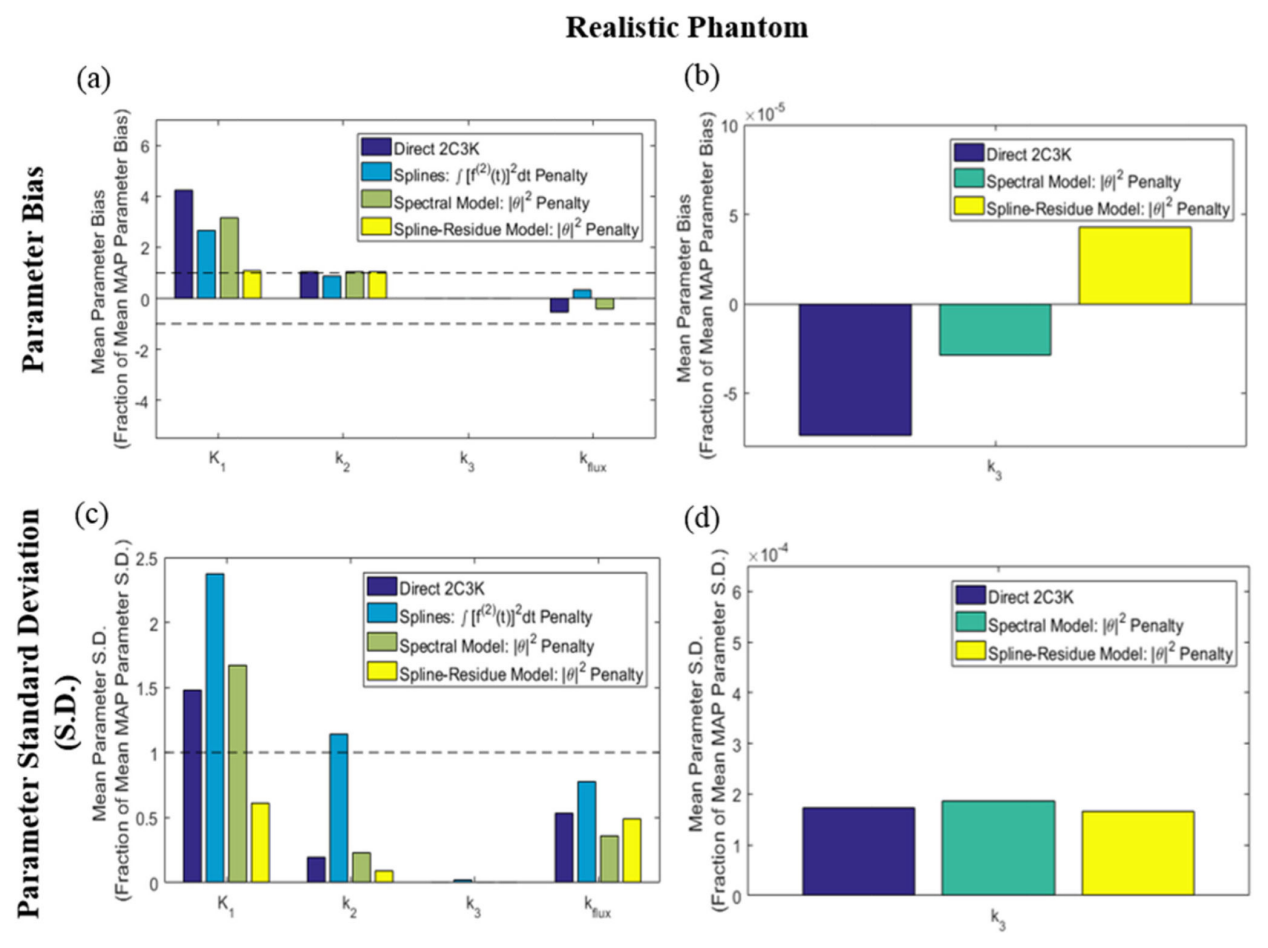
(a) Bias and (c) noise (standard deviation) of fitted parameter values in parametric maps derived from the 4D-PET reconstructed images of the realistic phantom, averaged over the entire parametric map of the image sub-region shown in figure 3(b). Values are plotted for each of the kinetic parameters as fractions of those obtained for the same parameter from analysis of conventional (non-4D) MAP-reconstructed images. Results for the *k*_3_ parametric maps obtained from the 2C3K, spectral and spline-residue model based 4D reconstructions are re-plotted on larger scales in (b) and (d) to make them visible.

**Figure 11:**
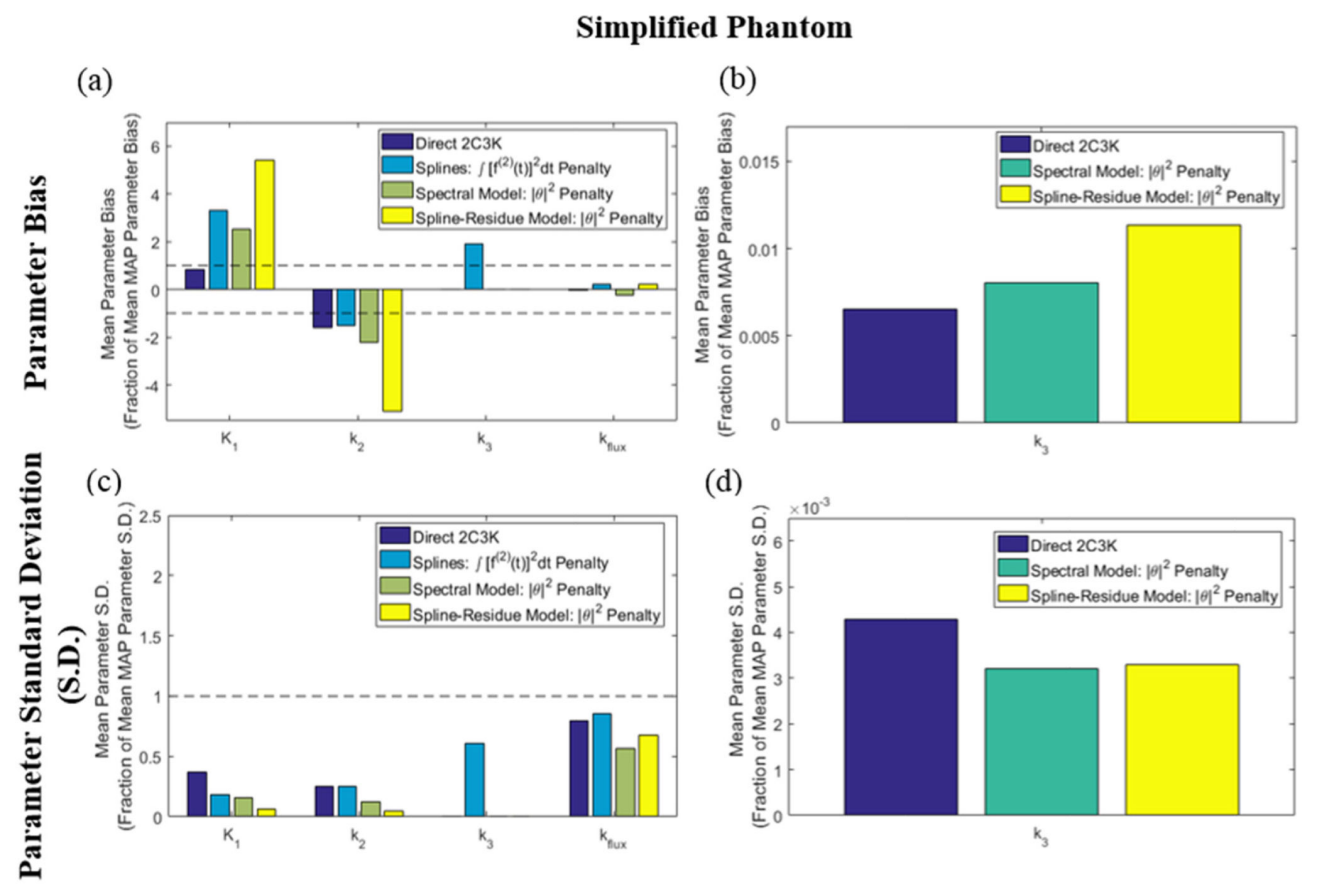
(a) Bias and (c) noise (standard deviation) of fitted parameter values in parametric maps derived from the 4D-PET reconstructed images of the simplified phantom, averaged over the entire parametric map of the image sub-region shown in figure 3(b). Values are plotted for each of the kinetic parameters as fractions of those obtained for the same parameter from analysis of conventional (non-4D) MAP-reconstructed images. Results for the *k*_3_ parametric maps obtained from the 2C3K, spectral and spline-residue model based 4D reconstructions are re-plotted on larger scales in (b) and (d) to make them visible.

**Figure 12:**
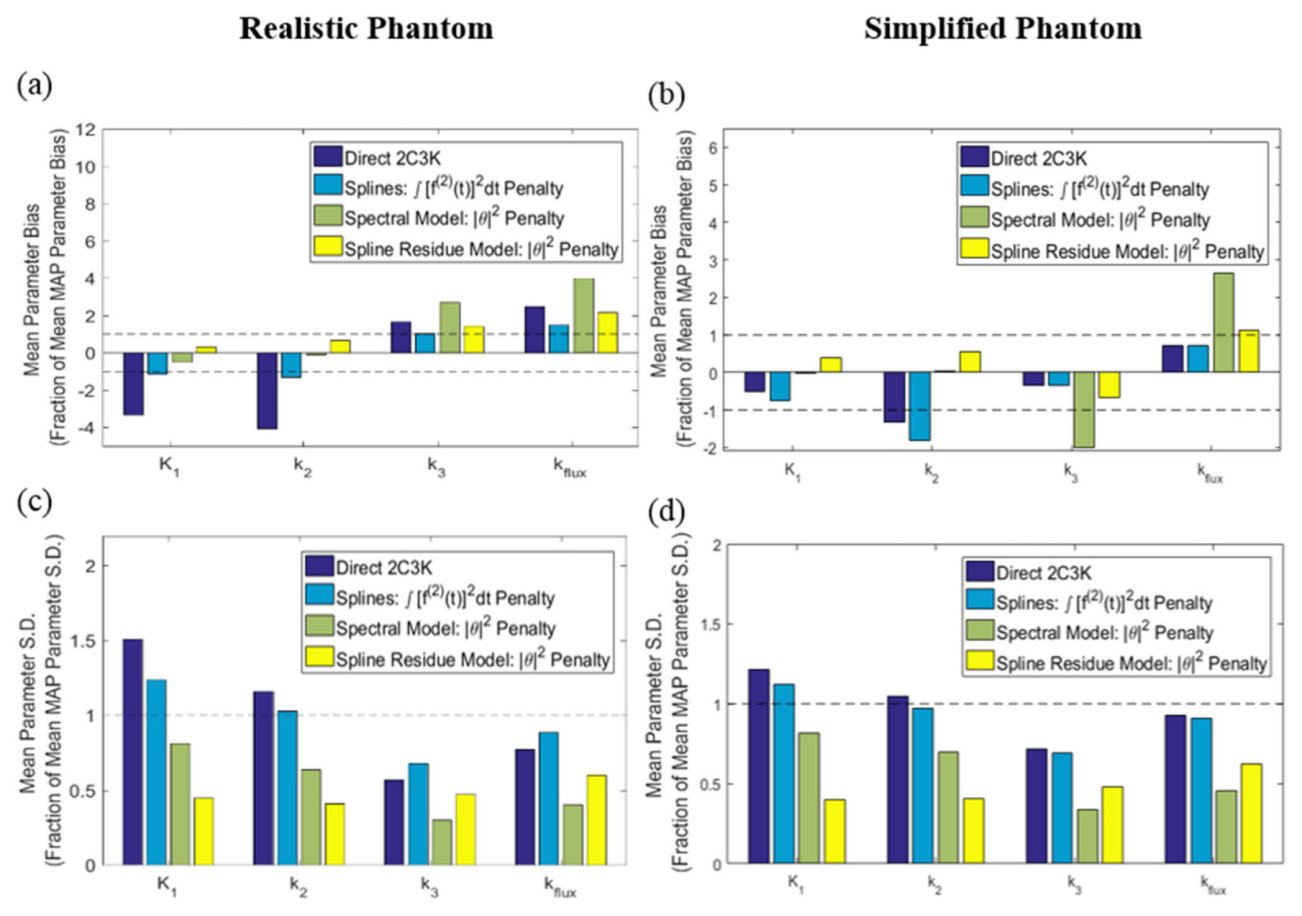
(a) and (b) Bias and (c) and (d) noise (standard deviation) of fitted parameter values in parametric maps derived from the 4D-PET reconstructed images of the realistic phantom, averaged over the hypoxic tumour region alone. For each of the kinetic parameters, the values plotted are fractions of those obtained for the same parameters from analysis of conventional (non-4D) MAP-reconstructed images.

The average normalised bias and noise (standard deviation) in 2C3K model parameters, for fits to the TACs of every voxel of the realistic phantom sub-region of figure 3(b) in images reconstructed using the different algorithms, is shown in figure 10 for the individual 2C3K rate constants and the composite *k_flux_* parameter. For each parameter, these values are normalised as fractions of the values achieved by conventional (non-4D) MAP reconstruction for the same parameter, thus showing the extent to which each nested-MAP reconstruction improves on the conventional MAP reconstruction for each kinetic parameter. Equivalent plots for the simplified phantom are shown in figure 11. Bias and noise values averaged over the smaller hypoxic tumour sub-volume alone are shown in figure 12 for the realistic and simplified phantoms.

For the realistic phantom it can be seen from figure 10 that for five of the eight combinations of bias/noise and kinetic parameters analysed, bias or noise averaged across the whole phantom was reduced more than 50% by using 4D reconstruction based on the spline-residue model rather than conventional (non-4D) MAP-reconstruction. Furthermore, for 5/8 combinations spline-residue 4D reconstruction produced better results than any of the other reconstruction algorithms studied, while spectral-based 4D reconstruction produced the best results for 2/8. Compared to conventional reconstruction, spline-residue-based 4D reconstruction did not notably increase bias or noise for any of the eight combinations, whereas the bias and noise in fitted *K*_1_ parameter values rose substantially above the conventional MAP levels when 4D reconstruction was carried out using the other temporal models. 4D reconstruction based on the 2C3K model generated the most biased kinetic parameters, and higher levels of noise than spline-residue-based reconstruction. From figure 12 it can be seen that for the hypoxic tumour region alone, the spline-residue- and spectral-based 4D reconstructions each achieved the best results for 3/8 of the bias/noise and kinetic parameter combinations analysed, and cubic spline-based 4D reconstruction the best results for 2/8.

For the simplified phantom, whose kinetics entirely follow the 2C3K model, 4D reconstruction based on the 2C3K model unsurprisingly achieved the lowest levels of bias for fitted 2C3K model kinetics parameters (see figures 11 and 12). This algorithm also achieved useful reductions in average noise levels for 2C3K parameter values throughout the simplified phantom compared to conventional MAP reconstruction, although not within the hypoxic tumour region. 4D reconstruction based on the spline-residue and spectral models achieved the greatest reductions in noise, but outside of the hypoxic tumour region the bias in fitted *K*_1_ and *k*_2_ values was larger for these algorithms than for conventional MAP reconstruction, particularly so for the spline-residue model.

## 4 Discussion

We hypothesized that 4D-PET reconstruction based on the linear spline-residue model might offer advantages for dynamic PET scanning of regions in which not all TACs are accurately described by the simple ‘2C3K’ irreversible two-tissue compartment model. In this study, we have compared results obtained using this proposed algorithm to those achieved using conventional (non-4D) MAP reconstruction, and 4D reconstruction based on adaptive-knot cubic splines and the spectral and 2C3K models. Working with a geometry based on thoracic anatomy, we calculated results for a ‘realistic phantom’ in which noise-free ground-truth TACs were represented by statistically acceptable fits of cubic splines and a ‘3C5K’ compartment model to TAC data obtained from a patient with stage IV NSCLC. We obtained further results for a ‘simplified phantom’ in which ground-truth TACs were represented by fits of the simple ‘2C3K’ compartment model, which did not describe the real data well.

For the realistic phantom, 4D reconstruction based on spline-residues generated less bias or noise in parameter maps of fitted kinetic values than did any of the other algorithms studied, in 5/8 of the combinations of bias/noise and kinetic parameters we analysed. Additionally, the spline-residue algorithm reduced bias or noise by over 50% compared to conventional (non-4D) MAP reconstruction in 5/8 combinations, and notably increased bias or noise in none. 4D reconstructions based on the 2C3K model generated the most biased kinetic parameters, and also generated higher levels of noise than did spline-residue-based reconstruction.

If anything, our analysis should favour 4D reconstruction based on the simple 2C3K compartment model. This is the model most commonly used in the literature to characterise FMISO kinetics (Wang *et al* 2009, McGowan *et al* 2017), and consequently the one we fitted voxel-by-voxel to TACs obtained from reconstructed images when characterising the accuracy and precision of the tracer kinetics in the images. Despite this, the parametric map results obtained from 4D reconstructions based on the 2C3K model were poorer than those from spline-residue- and spectral-based 4D reconstructions. Results obtained from cubic spline-based 4D reconstructions were also generally worse than those from the spectral and spline-residue-based reconstructions.

For the simplified phantom 4D reconstruction based on the 2C3K model performed much better, achieving lower levels of bias in fitted kinetic parameter maps than any of the other reconstruction algorithms studied, and useful noise reductions compared to the conventional MAP algorithm. Thus, the 2C3K-based 4D algorithm might be expected to provide good results in situations where the radiotracer kinetics are accurately described by the 2C3K model throughout the imaged field-of-view.

The results obtained for parametric maps largely concord with those of our more direct analysis of the accuracy and precision of reconstructed images (figures 6 and 7). For the realistic phantom, 4D reconstructions based on the spline-residue and spectral models produced higher quality images than reconstructions based on the 2C3K model or cubic splines. Across all time-frames and phantom regions, the quality of images reconstructed using the spectral model-based 4D algorithm was slightly better than that of images reconstructed using the spline-residue-based algorithm. However the spine-residue-based algorithm generated much higher quality images at early time-points, perhaps because it comprises far fewer basis functions and is therefore less likely to overfit data; and presumably this gain at early time-points led to the *K*_1_ and *k*_2_ parametric maps generated from spline-residue-based reconstructions being of a higher quality overall than those obtained from spectral-based reconstructions.

Images produced by linear model-based 4D reconstruction algorithms were much noisier when temporal roughness was not penalised (see figure 5). For the phantoms we studied, linear model-based 4D reconstructions produced less biased results than 2C3K-based 4D reconstructions at comparable or lower noise levels. However, it should be noted that this advantage only became apparent when temporal roughness penalties were built into the linear model-based reconstructions. Most linear model-based 4D-PET algorithms described in the literature do not incorporate such penalties, possibly because this makes the algorithms more complex. However, this may put them at a disadvantage when compared to 4D algorithms based on less highly parametrised non-linear kinetic models.

Algorithm convergence is not guaranteed when model fitting step (15) is instead accomplished via the penalized weighted least squares approach taken here. However, the results in figure 8 show that the nested-MAP reconstructions did approach convergence within 30 reconstruction iterations, consistent with observations of convergence reported by Matthews *et al* (2010) for 4D reconstruction algorithms using weighted least squares.

Allowing the $\gamma_{j}^{\prime }$ values, and thereby the objective function, to vary at each iteration could also affect convergence. To check this, for each linear model we re-ran each temporally regularized reconstruction (using the optimal penalty function for each model) with fixed $\gamma_{j}^{\prime }$ values, taken as those obtained in the final iteration of the corresponding reconstruction where $\gamma_{j}^{\prime }$ was free to vary. The results, plotted in supplementary figure S1 (stacks.iop.org/PMB/63/095013/mmedia), show that beyond 5 iterations the convergence of reconstructions obtained with fixed and varying $\gamma_{j}^{\prime }$ was identical.

*L*_1_ regularization (Λ (***θ***) = *|**θ***|) has been proposed for post-reconstruction fitting of the spectral model (Gunn *et al* 2002), with the aim of producing sparse solutions. This is useful if one intends to calculate physiologically relevant kinetic parameters directly from the ***θ*** values. However, we use linear models simply to produce smooth descriptions of the reconstructed TACs during 4D reconstruction, the resulting reconstructed images subsequently being analysed with a compartment model. Consequently, our priority was to use regularization to limit overfitting, and therefore we explored *L*_2_ regularization (Λ (***θ***) = *|**θ**|*^2^), which is specifically designed for this purpose and is more computationally efficient than *L*_1_ regularization.

The value of the spatial regularization parameter, *β*, was fixed at 0.1 in this study. This value was optimal for static reconstructions of a NEMA-like digital phantom using the conventional MAP algorithm, but might not be best for the nested-MAP reconstructions of the phantoms studied in this work. We therefore repeated the linear model-based nested-MAP reconstructions of the realistic patient phantom, using *β* values of 0.01 and 1. Figure S2 in the supplementary material shows the resulting iteration-by-iteration plots of image bias versus noise: for all the reconstructions *β* = 0.01 produced substantially noisier images, while *β* = 1 led to slightly less noisy but slightly more biased images than those produced with *β* = 0.1. Thus there appears to be little to gain, and much to lose, by using *β* values other than 0.1 for the algorithms and phantoms studied here.

We have focused on a phantom with kinetics taken from an FMISO dPET scan of an NSCLC patient, FMISO being of interest in oncology as a tracer of tumour hypoxia (Wang *et al* 2009, Cheng *et al* 2014, McGowan *et al* 2017). However most dPET studies use other tracers, particularly FDG. Examples of patient tissue TACs that were poorly described by non-linear kinetic models have been reported in the literature for tracers other than FMISO (O’Sullivan *et al* 2009, Matthews *et al* 2012), and so 4D-PET reconstruction based on the more flexible linear spline-residue and spectral models may be useful for a wider range of tracers than FMISO alone.

In another 4D-PET reconstruction algorithm proposed by Kotasidis *et al* (2014) for fields-of-view containing tissues with diverse kinetics, a ‘primary’ kinetic model of interest is initially fitted to voxel TACs, and then a more flexible ‘secondary’ model is fitted to the residuals in regions where the primary model fit is poor, thus limiting bias propagation from these regions. An open question, however, is what form the secondary model should take. The results of our study suggest that the spline-residue model would make a good secondary model in Kotasidis’ algorithm, since it is able to fit a wide range of TAC shapes while still suppressing noise.

Finally, O’Sullivan *et al* (2009) have shown that fits of a residue model based on splines provided better descriptions of TACs taken from previously reconstructed FDG-dPET brain image sequences than did fits of the 2C3K model, allowing more robust estimation of kinetic parameters such as *K*_1_, *k_flux_*, median radiotracer transit time and fractional blood. Thus spline-residue-based 4D-PET reconstruction may prove useful for direct determination of some physiologically relevant kinetic parameters, as well as being a means to limit noise amplification during reconstruction before kinetic analysis of the resulting smoother images using a compartment model.

## 5 Conclusion

Of all the reconstruction algorithms studied in this work, the proposed spline-residue-based 4D-PET reconstruction algorithm overall produced the highest quality (least biased or noisy) parametric maps of FMISO uptake kinetics for a phantom with a thoracic geometry and realistic FMISO uptake kinetics derived from those in a dynamic scan of an NSCLC patient. Specifically, reconstruction of images using this algorithm rather than the conventional (non-4D) MAP algorithm led to reductions of 50% or more in bias or noise for a majority of the combinations of kinetics parameters and bias/noise measures we analysed, and did not generate notably worse results for any combination. 4D reconstruction based on the simple irreversible two-tissue compartment model 2C3K produced the most biased parametric maps for this phantom overall, and generated notably higher levels of bias and noise in the *K*_1_ kinetics parameter than those obtained from conventional image reconstruction.

For a simplified phantom in which all ground-truth kinetics followed the poor descriptions of real data provided by fits of the 2C3K model, 4D reconstruction based on this model achieved the lowest levels of bias in fitted kinetic parameter maps. Thus, the 2C3K-based 4D algorithm can provide good results when the kinetics of radiotracer uptake throughout the field-of-view are described well by the 2C3K model.

When the underlying kinetics of tracer uptake in all imaged tissues are not well described by a simple compartment model, as for the FMISO kinetics studied in this work, it is advantageous to perform 4D-PET reconstruction using more flexible linear kinetic models, the spline-residue model proving the best of the models we studied. Temporal roughness penalties improve the performance of 4D-PET reconstruction algorithms based on linear kinetic models, the optimal penalty function depending on the linear model being used.

## Figures and Tables

**Table 1:** Weighted RSS errors for model fits to patient TACs calculated using leave-one-out cross-validation. The lowest RSS value for each tissue region is shown in bold. Fits that passed the Wald–Wolfowitz runs test at the 5% significance level are underlined.

Model	Healthy lung	Healthy spine	Hypoxic tumour	Normoxic tumour
2C3K	6.64 × 10^5^	5.65 × 10^5^	4.57 × 10^5^	3.94 × 10^5^
3C5K	4.53 × 10^5^	3.50 × 10^5^	**1.84 × 10^5^**	**1.31 × 10^5^**
Adaptive-knot cubic B-splines	**3.63 × 10^5^**	**1.97 × 10^5^**	1.87 × 10^5^	1.48 × 10^5^

**Table 2:** Mean signed parameter bias and standard deviation (S.D.) in *k_flux_* parametric maps of the realistic phantom, averaged over the sub-region shown in figure 3(b). The maps were obtained from image sequences reconstructed using the conventional (non-4D) MAP algorithm, and the 2C3K and linear model-based nested-MAP reconstructions (with their optimal temporal roughness penalties). Bias and S.D are expressed as percentages of the average ground-truth *k_flux_* value across the phantom sub-region. The lowest bias and S.D values are highlighted in bold and underlined.

Algorithm	Kinetic model fitted during reconstruction	Bias (%)	S.D. (%)
MAP	None	7.9	15.8
Nested-MAP	2C3K	−4.2	8.4
Nested-MAP	Adaptive-knot cubic B-splines (*∫* [[*f*^(2)^ (*t*)]^2^*dt* penalty)	2.6	12.1
Nested-MAP	Spectral model (|*θ*|^2^ penalty)	− 3.2	**5.7**
Nested-MAP	Spline-residue model (|*θ*|^2^ penalty)	**−0.1**	7.7

## Footnotes

Supplementary material for this article is available online
